# ‘I think writing is everything’: An exploration of the writing experiences of people with aphasia

**DOI:** 10.1111/1460-6984.12762

**Published:** 2022-08-05

**Authors:** Lindsey Thiel, Paul Conroy

**Affiliations:** ^1^ Leeds School of Social Sciences Leeds Beckett University Leeds UK; ^2^ Division of Human Communication Development and Hearing, School of Health Sciences University of Manchester Manchester UK

**Keywords:** aphasia, dysgraphia, writing, literacy, perceptions, experiences

## Abstract

**Background:**

Written communication has become an increasingly important part of everyday life in social, educational and professional spheres. The substantial increase in writing via the internet and mobile technologies provides both an opportunity for social engagement and distinct challenges for people with aphasia. Within the current literature there has been limited research into the lived experiences of people with aphasia of their writing difficulties and how these affect their ability to communicate.

**Aims:**

This qualitative study aimed to explore the experiences of people with aphasia of living with language‐related writing difficulties and the impact of these on their lives.

**Methods & Procedures:**

Eight people with post‐stroke aphasia and writing difficulties took part in semi‐structured interviews. The interviews were analysed using inductive reflexive thematic analysis.

**Outcomes & Results:**

Two themes were found in the data. The first theme was a *gradual and effortful improvement to writing*: Participants described how writing had improved since their stroke due to strategies and support, but they still found writing to be difficult and frustrating and described many barriers to writing. The second theme was the *importance of writing for fulfilling adult social roles*: Participants found writing to be important for communicating with family, friends and organizations, but their participation in society and self‐esteem and confidence were impacted by writing difficulties; reduced social roles meant reduced need for writing, but participants were still motivated to work towards writing goals.

**Conclusions & Implications:**

The findings demonstrate the emerging importance of writing skills for people with aphasia with respect to communication, well‐being, participation and inclusion in society, and carrying out social roles. They provide an insight into the process of improvement, including the difficulties, facilitators and barriers. Implications for speech and language therapy assessment and management are discussed.

**WHAT THIS PAPER ADDS:**

**What is already known on the subject**

- People with aphasia have difficulties with writing that can affect their ability to communicate. A small body of qualitative research has provided insights into individuals’ experiences of literacy difficulties. More research is needed to understand the writing experiences of people with aphasia to help design appropriate assessments and interventions.

**What this paper adds to existing knowledge**

- Participants experienced gradual and effortful improvement since their stroke. They felt negative about aspects of their writing, including speed, accuracy and range of vocabulary. Writing was facilitated through assistive technologies, spelling practice and support from others; barriers included technology, lack of time, stroke‐related symptoms and others’ lack of awareness about aphasia. Participants considered writing skills to be important, particularly for communication, carrying out adult social roles and participating in society, and were therefore still working towards goals related to everyday writing activities.

**What are the potential or actual clinical implications of this work?**

- This study suggests that speech and language therapy assessment should include interviewing participants about their activities, strengths, difficulties, facilitators and barriers in writing, and informal assessment of a range of functional writing tasks. Intervention should be tailored to the individual's needs. This should include meaningful activities that relate to functional everyday writing and, where appropriate, self‐management, compensatory technologies and group approaches, while making use of existing strategies identified by the individual.

## INTRODUCTION

Aphasia can have a profound impact on a person's life, activities, participation and social roles due to difficulties with spoken language, understanding, reading and writing (Papathanasiou et al., 2016). Qualitative research has explored the experiences and goals of people with aphasia and has provided important clinical insights which potentially indicate a framework for clinical management (Brown et al., 2010; Cruice et al., 2006; Grohn et al., 2014; Simmons‐Mackie & Lynch, 2013; Worrall et al., 2011).

Interviews conducted by Brown et al. (2010) examined how 25 people lived successfully with aphasia, focusing on the positive aspects of their recovery. The themes that emerged from participants’ descriptions were ‘doing things’, ‘meaningful relationships’, ‘striving for a positive way of life’ and ‘communication’. These findings support a holistic approach to aphasia management that explores the impact of aphasia on activities that are important to the client, including intervention that addresses everyday communication, participation in meaningful communicative activities, and supporting clients to maintain and build relationships with family, friends and other people with aphasia.

The goals of 50 people with aphasia were explored by Worrall et al. (2011), who conducted semi‐structured in‐depth interviews and then coded the responses using the International Classification of Functioning (ICF) (WHO, 2015). Their participants wanted to return to their pre‐stroke lives, to be able to communicate, to have access to information, to have speech and language therapy and other services that met their needs, to have control and independence as well as dignity and respect, to engage in social, leisure and work activities, to help others and to contribute to society and to have good physical health. Most of these goals linked to the activity and participation levels of the ICF, which demonstrates the importance of everyday activities for people with aphasia and the need for these to be considered in aphasia rehabilitation. The authors argued that aphasia services should be planned around the major components of the ICF and have ‘strong relationship‐centred, aphasia‐friendly goal setting’ as their focus (Worrall et al., 2011: 320).

Written communication has become increasingly important in recent years for participation in social and professional domains, particularly due to the increased reliance on internet and mobile technologies for communication (Deursen & van Dijk, 2010; Kjellen et al., 2017; Menger et al., 2016; Steyaert, 2002). Research into writing difficulties in people with aphasia (acquired dysgraphia) has focused on the cognitive and linguistic aspects of writing and the quantitative outcomes of writing therapies (Thiel et al., 2015). There has been limited exploration into the lived experiences of people with acquired dysgraphia, except for a small number of studies (Kjellen et al., 2017).

Parr (1995) investigated factors relating to functional reading and writing. A total of 20 participants with mild to moderate aphasia were interviewed about their premorbid and current roles, reading and writing activities, changes in roles, and coping mechanisms. She found a large variation across participants in their roles both before and after their stroke with a complex combination of reasons for role changes being given, including, for example: aphasia, loss of confidence, lack of money and motor problems. Moreover, a large range of writing activities were described, with none of the participants having the same combination of activities. Participants described different types and levels of support from family members, friends and technologies. This study demonstrated that reading and writing activities are ‘embedded in social, domestic and cultural patterns of behaviour and organization’ (Parr, 1995: 224) and emphasized the value of using interviews as part of initial assessments so that abilities and preferences can be established, and appropriate therapy goals can be set. Given the dramatic changes to purposes for writing since this paper was published due to technological advances, for example, increased use of social media, text messages and emails to communicate, more current evidence is needed to understand the reading and writing activities of people with aphasia and the strategies and support that they are drawing on.

A qualitative descriptive study by Kjellen et al. (2017) aimed to further improve understanding of the ‘insider's perspective’ of everyday literacy. A total of 12 Swedish‐speaking participants with mild to moderate aphasia (one participant had severe aphasia) took part in semi‐structured interviews. The overarching theme that emerged from the data was ‘literacy as an ongoing recovery process’, which consisted of two subthemes. Within the first, ‘changes in conditions for literacy’, participants described changes in reading and writing habits with constantly improving reading ability and slowly improving writing ability. The second subtheme, ‘facing expectations about literacy’, described how participants face expectations related to reading and writing, including their motivations, strategies and positive effects of practice. The participants described the ongoing process of recovery and the progress made, which demonstrated that people with aphasia can improve reading and writing skills, but that lifelong learning may be necessary.

These previous studies have provided important insights into the literacy experiences of people with aphasia. However, more research is needed that focuses more specifically on writing experiences, including current writing activities, strategies and support, barriers, as well as future goals and the impact of writing impairments on identity and social roles. Considering the broad range of writing activities and support described by Parr (1995), further exploration through interviews with different people with aphasia who are likely to have different lived experiences will contribute to and build on the existing literature. As well as participants having different dysgraphia symptoms and severities, the context and therefore the experiences of participants are likely to be different from those in the previous studies, due to factors such as the year that the interviews took place, previous therapy, and years post‐stroke.

Gaining more insights from participants on their writing activities, strategies, support and barriers can provide valuable information to speech and language therapists on the development of functional writing assessments and interventions that focus on activity and participation levels of the ICF (WHO, 2015), as the focus has largely been on impairment‐based assessments and therapy (Thiel et al., 2015). This study aimed to contribute to the literature through answering the following research question: What are the writing experiences of participants living with acquired dysgraphia?

## METHODS

### Design

This study used a qualitative design to explore participants’ experiences of living with acquired dysgraphia through semi‐structured interviews. As the aim was to understand and describe participants’ subjective realities, a constructivist paradigm was adopted, which assumes that there are multiple realities that are socially and experientially constructed by individuals, and that knowledge is subjective and socially constructed (Braun & Clarke, 2020; Brown & Dueñas, 2019; Guba & Lincoln, 1994; Lawton et al., 2018). In a constructivist paradigm the findings are perceived to be created through the interaction between the researcher and participant (Guba & Lincoln, 1994).

### Participants

Eight participants were invited to participate in the current interview study. All eight had previously taken part in in a previous study (Thiel et al., 2016) in which they were trained to use Co:Writer^®^, an assistive writing technology. Inclusion criteria for the previous study were that participants had to have acquired dysgraphia following a stroke; be at the chronic stage of their brain injury (i.e., post‐6 months); have sufficient visual acuity and motor ability for writing on a computer; and be monolingual speakers of English. Participants were screened for inclusion using writing subtests from the Comprehensive Aphasia Test (Swinburn et al., 2004). Before training commenced, to provide information on their language abilities, they were assessed on the Boston Diagnostic Aphasia Examination (BDAE; Goodglass et al., 2001), a comprehensive neuropsychological language assessment battery for people with aphasia, and the Pyramids and Palm Trees Test (Howard & Patterson, 1992), an assessment of semantic access from pictures. Participants’ demographic information and scores from these assessments are displayed in Tables 1 and 2. The participants had a broad range of types and severities of aphasia and acquired dysgraphia. At the end of the previous study, the eight participants were invited to take part in this study and all eight consented to take part.

**TABLE 1:** Participants’ demographic information and screen scores

		**Participants**							
		**1**	**2**	**3**	**4**	**5**	**6**	**7**	**8**
Age (years)		66	58	50	58	74	80	47	50
Gender		Female	Male	Male	Female	Female	Female	Male	Female
Education (years)		11	12	16	11	11	9	10	10
Occupation		Retail manager	Regional retail manager	Building surveyor	Personal assistant	Administrator	Factory supervisor	Factory worker	Care manager
Event		CVA	CVA	CVA	CVA	CVA	CVA	CVA	CVA
Years;months since stroke		18 years	2;10	6;8	6 years	4;6	19;3	3;11	4;2
CAT scores (no. letters correct)	Copying	27/27	27/27	27/27	27/27	25/27	26/27	27/27	27/27
	Written picture naming	7/21	13/21	19/21	17/21	13/21	17/21	18/21	18/21
	Writing to dictation	12/28	5/28	17/28	6/28	13/28	16/28	26/28	24/28
	Written picture description	3	6	2	15	4	1	8	22

*Note*: CAT, Comprehensive Aphasia Test (Swinburn et al., 2004).

**TABLE 2:** Participants’ BDAE and PPT raw scores

	**Participants**								
	**1**	**2**	**3**	**4**	**5**	**6**	**7**	**8**	**Maximum score**
Fluency	18	16	11	3	13	4	21	17	21
Conversation	7	6	6	3	5	6	7	7	7
Auditory comprehension	25.5	28	20	21	30	27	24	30	32
Articulatory agility	5	4	4	4	3	2	7	5	7
Recitation	3	4	4	0	2	4	4	4	4
Repetition	4	6	5	3	3	4	7	5	7
Naming	28	27	30	1	20	22	27	31	37
Reading	16	27	36	20	28	31	35	37	39
Writing	47	58	58	52	40	43	63	66	73
PPT^a^	45	50	52	51	49	46	43	48	52
Aphasia severity	Mild	Moderate	Moderate	Severe	Moderate	Severe	Mild	Mild	
Aphasia classification	Anomic	Anomic	Mixed non‐fluent	Mixed non‐fluent	Conduction	Broca's	Anomic	Conduction	

*Note*: BDAE, Boston Diagnostic Aphasia Examination: Short Version (Goodglass et al., 2001); PPT, Pyramids and Palm Trees Test (Howard & Patterson, 1992); ^a^Cut‐off = 49/52.

### Procedure

This study was approved by the Health Research Authority National Research Ethics Committee North West Greater Manchester South (reference number 12/NW/0558). Participants were approached in final sessions of the previous study. They were given a participant information sheet and were told about the study verbally. They had an opportunity to ask questions and to consider whether they would like to participate, before being asked to sign a consent form. Semi‐structured interviews were conducted by the first author (L.T.) with all participants to explore their subjective experiences of their dysgraphia. The interviews took place between May and October 2014. A topic guide with predefined initial questions was used to structure the interview (see Appendix A). This was developed with support from an experienced qualitative aphasia researcher, external to the study (M.C.). Questions were formulated in such a way that it was likely they could be understood by the participants with the most severe language impairment. Further probe questions were asked depending on responses given by the participant. Initial questions were open‐ended as is recommended for qualitative research (Patton, 1987). However, as open‐ended questions can be difficult for people with aphasia to answer, many of the probe questions were then closed to facilitate participation (Luck & Rose, 2007). Interviews were 60–90 min in length, were single interviews and took place in participants’ homes. Although, all participants lived with a partner, only L.T. and the participant were in the room during the interview. The interviews included Part A (reported here) and Part B in which participants were asked about their experiences of taking part in the previous therapy studies. The findings from Part B will be analysed and reported separately. Sessions were video‐recorded to capture verbal and non‐verbal responses. Non‐verbal responses were described by the interviewer during the interview.

Due to the participants’ aphasia, L.T. used supportive conversation techniques to facilitate participants in providing responses. She acknowledged that she could therefore not remove herself from the interview process and that her involvement and her relationship with the participants, as the therapist in the previous study, may have influenced their responses. Participants were encouraged to provide answers in any way that they could, for example, by talking, pointing at pictures, writing, drawing pictures or gesturing. To facilitate communication during the interview, ‘communication ramps’ (Simmons‐Mackie et al., 2010) were provided. Picture cards were used to introduce and aid comprehension of each question. Furthermore, participants were given a scale to help them to describe how they view their ability in writing/computer skills or their opinion of therapies, etc. This was only provided if they had difficulty answering a question, so that participants were encouraged to describe their experiences in their own way, wherever possible. A timeline was provided to aid comprehension and answering of questions about different points of time. A written list of emotions was prepared before the interview so that alternatives could be given to the participant if they are unable to find words to express their feelings. The interview schedule was piloted with one participant. This was then watched back and discussed with an experienced qualitative aphasia researcher external to the study (M.C.), to develop L.T’.s skills in interviewing people with aphasia.

### Researcher characteristics and reflexivity

The first author (L.T.), who conducted the interviews, was a speech and language therapist and PhD student at the time of the interviews. Before participants were interviewed, L.T. had conducted a study with the same group of participants in which she had trained them to use an assistive writing software and evaluated the outcomes. Six of the participants had also taken part in a previous spelling therapy study that had been led by L.T. She had therefore been working with the participants for at least a year previous to the interviews. She was working as a senior lecturer in speech and language therapy when analysing the data. The data were thus viewed through the lens of a speech and language therapist who had an established therapeutic alliance with the participants (Lawton et al., 2018). She had experienced their progress through therapy and their challenges with writing and had been invested in their writing improvements. Interpretation of the data will have been influenced by L.T’.s relationship with the participants and knowledge of their experiences through previous conversations, assessments and therapies. L.T. therefore engaged in reflexivity through internal reflection and having open discussions with her supervisor (P.C.) at each stage of the research process, including during the interviews, transcription and analysis. The second author, P.C., is a senior lecturer in speech and language therapy and supported L.T. at each stage of the research project but did not have contact with the participants or directly analyse the data.

### Analysis

The interviews were transcribed verbatim by the first author (L.T.). Comments on other methods of communication were also included into the transcription. Interview data were analysed by L.T. using an inductive reflexive thematic analysis (Braun & Clarke, 2020; Braun & Clarke, 2006), an analytical approach of identifying patterns within data that has often been used in to explore the perceptions and experiences of people with aphasia (e.g., Brown et al., 2013; Lawton et al., 2018; Mumby & Whitworth, 2013; Tregea & Brown, 2013; Young et al., 2012). L.T. was new to qualitative research but had guidance and support from her supervisor (P.C.) with the analysis. In the initial stages of coding and theme development, semantic (surface‐level) meanings were captured, but as the analysis developed, latent, more conceptual, meanings were identified. The following iterative analytical process, outlined by Braun and Clarke (2006: 17–24), was followed:

- Phase 1: Familiarizing yourself with the data: Transcripts were read and re‐read and initial ideas about patterns were noted.
- Phase 2: Generating initial codes: The data were coded inductively, which involved working through each transcript and identifying semantic or latent meanings. The coding was performed manually with codes added as comments to the transcripts.
- Phase 3: Searching for themes: The codes were sorted by examining them for patterns. They were copied into a new document and those that were related in meaning were reorganized into lists of candidate themes and subthemes.
- Phase 4: Reviewing themes: This phase involved an iterative process of refining the themes and subthemes while reviewing and considering whether they reflected the coded data extracts and the data set as a whole. Negative cases were also sought. Some initial themes were rejected, while others were merged or divided.
- Phase 5: Defining and naming themes: This phase involved defining and refining themes and writing summaries to understand the essence of each theme. In this phase, theme names developed from being descriptive to more interpretative.
- Phase 6: Producing the report: This phase included the final analysis and write‐up of the ‘analytic narrative’ (Braun & Clarke, 2006: 93). Anonymised extracts from the data were selected to demonstrate the themes and subthemes.

### Rigour

Trustworthiness was enhanced in this study by ensuring through piloting that questions were accessible and understandable by the participants with aphasia. L.T. watched a video of the first interview to reflect on how well she used her interpersonal skills to support the participant's communication. An audit trail was kept throughout the analytical process. Verbatim quotations were used throughout the results section to illustrate themes, and findings have been compared with the broader literature. A reflexive approach was adopted with the context, the participants and the background of the researcher being described thoroughly. Finally, negative cases were analysed and described.

## RESULTS

**FIGURE 1:**
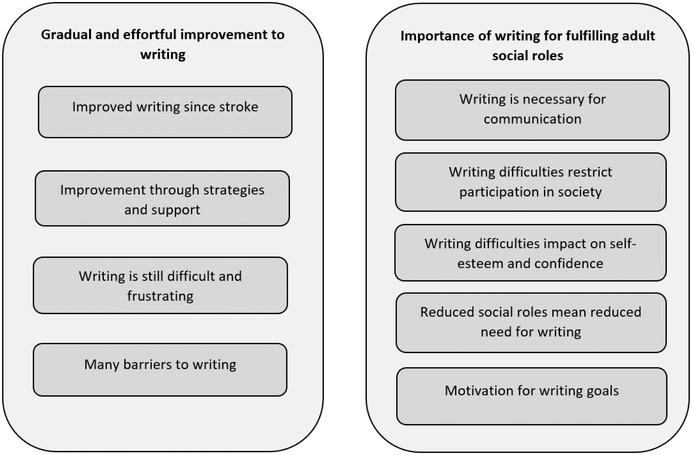
Themes and subthemes

The themes that were identified in the data are displayed in Figure 1. Table 3 shows the relationship between example interview extracts, codes, subthemes and themes.

**TABLE 3:** Development of themes with example interview extracts

**Interview extract**	**Codes**	**Subtheme**	**Theme**
but stroke slow down improving improving slow hell frustrating (participant 3)	Writing is improving Slow frustrating progress Gradual changes to writing	Improved writing since stroke	Gradual and effortful improvement to writing
It is getting better (participant 2)	Writing is improving		
I think like I say it's better but I need to do it all the time but this is it because three years nearly and I wouldn't be able to text properly but now I think it will be easier all the time now and quicker (participant 2)	Writing was difficult Writing is improving Writing is getting quicker		
I think the spell seem to come back gradually do you know what I mean gradually (participant 7)	Gradual changes to writing Writing is improving		
Participant 3: presentation powerpoint better better student student writing L.T.: ah yes so this is the student that helped you at the support group Participant 3: yeah yeah L.T.: to practise writing Participant 3: yeah yeah	Student at stroke group helped with writing	Improvement through strategies and support	
emails better and and software think so practice (participant 3)	Software helps with writing Writing got better because of practice		
well you lot really started me but my friend come every day because she was worried she she think I was going to a bit she she thought I was going to be used to but then she realised so what she can do she help me as well (participant 8)	Friend continued with practice when therapy stopped		
I know it's very easy but when you've got obviously aphasia but it's the writing, say I'm going write a Christmas card and erm I thought well I'll do it but I have to, I have to the name and happy birthday or Christmas and I have to [gestures writing with a pen] oh it's so hard and I'm like oh [puts hand to head act acts exhausted] (participant 1)	Writing is difficult Writing is exhausting	Writing is still difficult and frustrating	
how do you feel about your writing? Participant 6: er [holds up right arm and looks at it and then let's go of it to show how it weighs her down] L.T.: right ok yeah Participant 6: yeah L.T.: so because of your arm Participant 6: [touches her arm] L.T.: how about when you write, so you write with this arm usually [pointing at left arm] Participant 6: yeah L.T.: how do you feel about writing with this arm? Participant 6: [makes ‘ah’ sound as if exhausted or frustrated]	Hemiplegia barrier to writing Trying to use non‐dominant hand to write	Many barriers to writing	
Participant 4: menu L.T.: yeah did you write down what you wanted? Participant 4: yeah wonderful L.T.: so you wrote down a list for the waitress? Participant 4: yeah [smiles] L.T.: and did she understand? Participant 4: yeah wonderful	Writing helps to communicate	Writing is necessary for communication	Importance of writing for fulfilling adult social roles
L.T.: and then there's some messages. Is that one from your son? Participant 5: yeah I read them and I don't, I read them I can't I don't write them L.T.: ah ok Participant 5: yeah L.T.: so you mean you don't reply? Participant 5: yeah L.T.: yeah ok is that because it's so difficult? Participant 5: yeah	Difficult to reply to text messages	Writing difficulties restrict participation in society	
and I feel a bit conscious because fully grown woman can't spell and I think but you have to get over that, but it's very hard very hard (participant 1)	Can't spell Feeling self‐conscious because of spelling difficulties	Writing difficulties impact on self‐esteem and confidence	
I could do writing I do writing is very good erm I don't have to do it anymore (participant 5)	Don't have to write anymore Happy with writing	Reduced social roles mean reduced need for writing	
but now Fred said don't worry about the money so he does it so yeah so I've got used to it now and I'm happy because I don't have to work (participant 8)	Got used to husband doing the finances Don't have to write anymore		
L.T.: yeah yeah so what things would you be able to do if you could write sentences. What would that help you to do? Participant 2: erm if it would be a case of erm bills and things like that	Wants to write so that can pay the bills	Motivation for writing goals	
I didn't practise enough [xxx] my spelling now practice practice practice all the time [xxx] (participant 5)	Currently practising		

In this section the themes and subthemes will be described. Within excerpts from the interview transcripts, the researcher who conducted the interviews is labelled with the initials L.T., and participants are referred to with numbers 1–8. Pseudonyms are used for all names referred to in the transcripts.

### Theme 1: Gradual and effortful improvement to writing

The theme ‘Gradual and effortful improvement to writing’ captured participants’ experiences of the process of improvement to writing, which included improved spelling but also improved ability to complete writing activities through compensatory methods. Participants described improvements since their stroke (subtheme 1), and also the strategies and support that facilitated these improvements (subtheme 2). Despite gradual improvements, participants still found writing to be difficult and frustrating (subtheme 3) and described many barriers to their progress in writing (subtheme 4).

#### Subtheme 1: Improved writing since stroke

The participants looked back to their writing skills immediately after their stroke and described them as much worse than they were at the point of the interviews, which was between 2 and 19 years later. In most cases, participants barely ever, or never, wrote in the early stages post‐stroke: ‘I couldn't read and erm my spelling was horrible’ (participant 8); ‘bad bad bad’ (participant 4).

They had experienced a slow and gradual process of improvement to writing skills: ‘but stroke slow down improving improving slow hell frustrating’ (participant 3); ‘something erm so basically it is it is obviously better it's erm slow slow but erm better all the time’ (participant 2); ‘I think the spell seem to come back gradually do you know what I mean gradually’ (participant 7). At the time of the study, most participants wrote frequently, as participant 2 described, ‘do it all every day’.

The participants found that writing improved with practice:

I think like I say it's er better but I need to do it all the time but this is it because three years nearly and I wouldn't be able to text properly but now I think it will be easier all the time now and quicker’ (participant 2).

#### Subtheme 2: Improvement through strategies and support

Participants showed a strong awareness of factors that had helped them in writing and each participant was supported by a range of individual factors. They had each discovered supportive strategies that included using a dictionary to check spellings, converting sounds to letters, writing in short sentences or slowing down to increase intelligibility. As participant 8 explained: ‘I write I could understand but not Fred my husband can as long as I slow it down.’

They also made use of technologies to support writing. Some found that there were general advantages to writing on a computer instead of writing on paper. For example, participant 3 described how he could structure his writing more easily on a computer, whereas participant 8 found that using a computer keyboard helped with her spelling:

because I can see it erm I think it when I got to write it I it's not like on the tablet you you got to write yourself so I know e but but in my mind I've got to think e [draws an ‘e’ with their finger].

Other functions on tablets and laptops helped with writing activities. For example, participant 5 made use of the direct link to the email app on her apple computer to avoid having to search for it by typing.

Participants used apps to compensate for their spelling difficulties. In most cases, apps were discovered by individuals themselves or were recommended by friends and family members. Participant 2 described how he had been introduced to apps by other stroke patients in hospital communication groups, while participant 1 was thrilled to have discovered voice recognition software on her iPad. For some participants, apps had an extremely positive impact on their written communication. Participant 1 spoke throughout her interview about how, through using voice recognition software, she could now write about anything:

honestly it's a godsend, honestly because before Frank was with me and can you spell this, can you spell and he said you need to practise more and er well I have and it's so hard […] I can talk to the tablet and it does it for me.

However, interestingly, she commented on this approach in a slightly critical way, describing it as ‘lazy’ or taking an easy option: ‘and er it's very lazy I know that but with the got stroke and erm aphasia it's so hard to you know really get, I do the easier way out’.

The participants found that other people also supported them with writing or using technologies for writing. This included partners, family members and friends. Participant 5 had support from her husband in writing emails: ‘yes the spelling it is he checks erm the er grammar grammar was is use a comma’. Participant 8 described how speech and language therapists ‘started her off’ with writing and then her sister and friends continued this with her:

well you lot really started me but my friend come every day because she was worried she she think I was going to a bit she she thought I was going to be used to but then she realised so what she can do she help me as well.

Participants felt that their writing had improved because they had practised, through copying words into a notebook from the dictionary or food packaging, or through using computer therapy software (React 2 and StepByStep) to practise spelling alongside other language skills, as participant 4 wrote: ‘step by step—Aphasia software excellent’.

Participant 3 attributed improvements to increased effort, support from a student at his support group but also spelling therapy (in the previous study), which had helped with writing through constant drilling. He compared the therapist to a woodpecker: ‘yes erm […] woodpecker [bangs on table] come on’.

#### Subtheme 3: Writing is still difficult and frustrating

Despite improvements, participants were still generally not happy with their writing. Participant 5, in particular, felt very unhappy with her writing skills, repeatedly describing them as ‘rubbish’. The participants also found writing to be extremely effortful: ‘might be better for me but I do it very slowly but you know I get by but the spelling is so hard and also it's the pain in my life’ (participant 1).

The participants provided more specific details on the aspects of their writing that they were unhappy with. This included not being able to do joined‐up handwriting, messages being too short, producing too many spelling errors, not being able to ‘get the words out’, writing too slowly, not understanding their own writing, not being able to think of what to write, or getting words muddled up. Participant 8, who had relatively mild difficulties compared with other participants, felt self‐conscious about her short messages and limited range of vocabulary. She was aware of how her messages looked compared with those of people without aphasia: ‘my writing is only short […] not like you’.

Although participants focused most on their writing difficulties, they also acknowledged that they had some strengths. For example, participant 8 felt ‘ok’ with text messages, emails and shopping lists. Participant 4 felt happy with her ability to write a greeting card. Participant 2 felt that he was able to recognize the correct spelling when he saw it. Participant 3 found writing single words easy and felt happy with writing emails if he did this slowly. For other participants there seemed to be an acceptance of their current writing ability: ‘I can get by, you know what I mean’ (participant 7).

#### Subtheme 4: Many barriers to writing

Participants’ improvements to writing skills were made in the face of multiple barriers. One challenge for participants was people's lack of awareness of aphasia:

they one letter and ah I can't spell and I thought I'd love to say ok I'll tell you why I can't er spell and they would ah oh so it's […] they don't know it's aphasia they no idea no why you don't need to but people don't know. (participant 1)

As participant 1 later explains, an additional difficulty was that people do not appreciate the level of support that she needed for writing a message: ‘she's not really she's not really getting it that I can't spell and she think oh you can but your text was lovely, you say yeah but I had to copy that’.

Another barrier for the participants was the amount of time that was needed to write accurately, as they recognized that they made more errors when they were busy and rushing: ‘not very good because erm I've been busy really’ (participant 8); ‘yeah it's just rushing that's all [laughs]’ (participant 7). Participant 8 described how distractions can impact on her writing: ‘it's when I know what I need to say but if er someone speaks to me it goes’.

Participants shared their insights into linguistic factors that made writing difficult. Sentences, abstract words (e.g., politics), homophones (e.g., ‘so’, ‘sew’), small (function) words (e.g., ‘of’), and longer words were all highlighted as difficulties for the participants: ‘you know like A or pen or whatever it's one word isn't it? but the sentences are completely different’ (participant 2).

The participants found that a further barrier to their writing was their own mood, mental health or fatigue. Participant 5 explained that her depression had an effect on how often she wrote. Participants’ writing and typing were additionally impacted by other stroke‐related disabilities including cognitive, visual and motor difficulties. Participants 4–6 all had a right‐sided hemiplegia and had learnt to write and type with their left hand, which added an extra difficulty to writing. Finally, some described their own lack of motivation or practice as a barrier: ‘I didn't practise enough (.) I didn't practise enough’ (participant 5).

### Theme 2: Importance of writing for fulfilling adult social roles

Throughout the interviews, participants expressed their feelings about the importance of writing for fulfilling adult social roles. Writing was considered important by most of the participants, as participant 1 stated: ‘I think writing is everything.’ Participants found writing to be important for communicating with family, friends and organizations, which is described in subtheme 1. Subthemes 2 and 3 capture how participation in society and self‐esteem and confidence were impacted by writing difficulties. Subtheme 4 shows that in contrast to the comments about writing having been important, the participants also discussed their current lack of opportunity for writing, and therefore a reduced need for writing skills, which was strongly related to their current social roles. Subtheme 5 captured the fact that participants were still motivated to work towards writing goals, which provided further support for the importance that writing had within their lives.

#### Subtheme 1: Writing is necessary for communication

The participants described the importance of writing for communicating with others, which helped them to fulfil particular social roles, for example, when asked about how writing is important to her, participant 5 described how writing is important for being a mother as she can communicate with her grown‐up children by text message: ‘I can't explain this, different ways important things mother a mother.’

Participants used social media and wrote text messages and email messages to friends and family members, usually to make plans:

saying that I'll do if I meet my friend I'll say Sally I'll meet you pub at ten o'clock or what other where where if I said to Yvonne I'll meet you at the pub at ten o'clock ten o'clock or maybe go for tea I write it all down. (participant 8)

They also reported writing to companies and charities, for example, to book things or to organize voluntary work.

Participant 8 who had a relatively mild writing impairment, imagined how difficult communication would be if she had not regained her writing skills within speech and language therapy:

oh god thank god you helped me erm my sister, imagine not saying my sister hello how are you and like we go on holiday. Imagine not doing that. It's bad enough not to see it but without writing it it means horrible. I know it's, some people can't but thank god erm I had the (.) you lot coming every day.

Some found writing to be an important mode of communication due to their spoken language or speech impairments. Participants 3 and 8 described how they could now communicate through text message, as they find it difficult talking on the phone: ‘even now I'm I I write things more than I can erm use the phone’ (participant 8). Participant 4, whose dominant mode of communication was writing single words in her notebook, due to her severe aphasia and apraxia of speech, described how she was able to write down what she wanted to eat in a restaurant and that she felt pleased when the waitress understood:

Participant 4: menu

L.T.: yeah (..) did you write down what you wanted?

Participant 4: yeah wonderful

L.T.: so you wrote down a list for the waitress?

Participant 4: yeah [smiles]

L.T.: and did she understand?

Participant 4: yeah wonderful

For participant 3, writing on his whiteboard had become an effective means of augmenting verbal communication: ‘spelling erm whiteboard sentence […] yeah crucial’.

In contrast to the other participants, participant 7, who despite being keen to take part in the study, expressed that he had never written much and was not intending to write much in the future: ‘just something that doesn't interest me’.

#### Subtheme 2: Writing difficulties restrict participation in society

The participants spoke about activities and social roles that they were no longer able to carry out due to their writing difficulties. This highlighted the importance of writing skills as their impairments impacted on their ability to participate in society.

For example, participant 2 described his difficulty with writing a reply to a letter about jury duty:

I could be able to do that you know whereas you know last week I had a letter er jury service. Now obviously you know I could have done that but in the end I had to go to the citizen's advice […] you know but I could've erm basically with the er letter the form I'm alright with that in terms of yes or no or my name and all that but then obviously I had to say why and I don't but was not why the jury service I could go and I'd like it to go.

He also felt that he would not be allowed to do jury duty, despite wanting to, because of his aphasia and literacy impairments: ‘basically they will say that I you know can't because I can't read or write’.

Participants described other activities that their writing difficulties impacted on such as paying the bills and filling in forms: ‘and you have to fill the form in and Frank wasn't there so I thought my god’ (participant 1). Participant 5 described how impaired spelling restricts her from responding to text messages from her son, writing emails and using the internet more generally: ‘yeah I read them and I don't, I read them I can't I don't write them’.

#### Subtheme 3: Writing difficulties impact on self‐esteem and confidence

Writing difficulties impacted the participants’ self‐esteem and confidence, which indicates their perception of writing as being a skill that adults are expected to have, in order to fulfil various adult social roles. Participant 4 described feeling depressed and angry for the first year after her stroke because of her aphasia and writing difficulties. Other participants described feeling stupid, self‐conscious or embarrassed when people saw that they could not spell.

Participant 1 described how she felt when filling out a form at a general practitioner's reception desk: ‘and I feel a bit conscious because fully grown woman can't spell and I think but you have to get over that, but it's very hard very hard’. She found writing to be more daunting because of her age: ‘when you when you're little you have practised and then it clicks you know writing and erm spelling but when you're older like forty odd you know it's so daunting’.

#### Subtheme 4: Reduced social roles mean reduced need for writing

Although most of the participants considered writing to be important for communication and participation in society, they had less need for writing in their lives since their stroke or retirement, due to a change of social roles and responsibilities: ‘I could do writing I do writing is very good erm I don't have to do it anymore’ (participant 5); ‘yes but now I'm not using it because I don't need to use it and erm it's still there in my brain if I need it but even now I don't need to do it’ (participant 8).

One of the reasons included for not having to write any more was due to no longer working in paid employment since their stroke. Most participants described how writing was important within their professional roles before their stroke; reports, timesheets and formal letters had been frequent activities. Writing was formerly central to participant 4's work life as a personal assistant, so some writing activities were no longer necessary when she stopped working:

Participant 4: [starts writing then gestures for the dictionary and then finds and writes ‘retirement’]

L.T.: right yeah ok cos you're in your retirement

Participant 4: yeah

L.T.: so you mean because of that writing isn't so important?

Participant 4: yeah

Participant 8 viewed the lack of writing as a partly positive change. She remembered working too hard as a care manager, which involved writing, whereas since her stroke she has had more time for socializing: ‘I used to think this was my life [pointing at paper].’

Another reason for not writing was not having anyone to write to: ‘I would like to do that but even saying that who would I write to?’ (participant 8). Some participants also described how they now had less need to write because partners had taken over any roles that required this:

erm yes but I I can't things like this erm it's too much for me to do the bills, Fred does the bills even though he lets me know what's what […] me and Fred used to do half and half’ (participant 8); ‘and Clare talk and erm email dates and times tee times. (participant 3)

Participant 8 reflects that her partner taking over these administrative tasks could be a blessing: ‘but now Fred said don't worry about the money so he does it so yeah so erm I've got used to it now and I'm happy because I don't have to work’.

#### Subtheme 5: Motivation for writing goals

Despite the change in participants’ social roles and reduced need for writing, they were still motivated to work towards specific individual writing goals, which emphasized the importance that they placed on their writing skills. These were varied and included writing poems, stories, text messages, greetings cards and letters, paying the bills and using Twitter. They aimed to reach their goals by continuing to practise their writing, including completing specific tasks such as email writing, or copying out single words or sentences into a notebook. Some participants planned to continue using the assistive writing software that they had been trained to use within the previous study, as they found that this supported their writing. Participant 4 planned to explore different compensatory technologies for writing. Participant 1 described how she would like for her writing to be how it was pre‐stroke, although recognized that this was an unlikely scenario:

and it's even nine nineteen years still erm really can't have feel it's still not perfect, I'm I always say I'm why you want to say that you can't you do it but you can't be perfect but I want that because I was like that before so it's and I want I want to be normal […] I want and I know I can't but I can dream.

## DISCUSSION

Eight participants with mild to severe aphasia took part in interviews about their experiences of writing. Data were analysed using inductive reflexive thematic analysis and the following themes were identified: Gradual and effortful improvement to writing, and Importance of writing for fulfilling adult social roles. These themes will be discussed in the context of the existing aphasia literature, and then the implications for clinical practice will be considered.

### Gradual and effortful improvement to writing

Participants’ writing abilities had improved since their stroke and were still improving, but changes were gradual and were associated with effort and frustration. This parallels the findings of Kjellen et al. (2017: 581) whose participants described that their writing had been ‘strongly impaired’ post‐stroke, had experienced positive changes since then but that this improvement happened slowly. In the current study, spelling practice was considered to be an important factor for improvement, either through using aphasia therapy software or copying out words. The literature has shown that self‐managed computerized speech and language therapy can lead to significant improvements to word‐finding in people with aphasia (Palmer et al., 2019), and that spelling practice which involves copying and recalling words can improve single‐word spelling (Thiel et al., 2015). The effort and frustration described by participants is unsurprising when findings from the spelling rehabilitation literature have demonstrated that gains from spelling therapies are often not generalized to functional writing (Thiel et al., 2015), and provides an argument for additionally using compensatory strategies to support functional writing.

Participants used a diverse range of strategies, particularly assistive writing technologies, to support everyday writing. Research has shown that using these assistive writing technologies can improve performance on functional writing tasks (Marshall et al., 2019; Thiel et al., 2016). Parr's (1995) participants used strategies such as drafting, proofreading and editing; using a dictionary; using memory buttons on phones, and using credit cards and cash dispensers to avoid the need to write cheques. In contrast to this study, none of her participants described using a computer to support everyday writing, which is unsurprising given that there were fewer technologies available to support writing at that time. Kjellen et al. (2017) found that their participants used some similar assistive technologies to the participants in the current study. The findings in this study have built on those from Parr (1995) and Kjellen et al. (2017) by providing insights into participants’ attitudes to using technologies (e.g., ‘it's very lazy’) and the positive effects of these technologies on their everyday writing activities (e.g., emails better and and software think so practice).

Participants had usually discovered strategies by finding them by themselves or being introduced to them by friends, family or others with aphasia. Furthermore, participants were generally aware of the impact of support from others, particularly family members and friends, on their ability to write. This reflects the findings of Grohn et al. (2014) who reported that an important factor for their participants in living successfully with aphasia was support from family, friends as well as rehabilitation services. These findings also provide support for group therapy, where participants can share ideas and learn from each other (Simmons‐Mackie & Elman, 2010). Interestingly, in this study when discussing the people who had supported them, speech and language therapists were rarely mentioned. This may be because it had been a long time since participants had been discharged from therapy. Alternatively, it may be the case that writing goals are not always a priority for clients or therapists in the first few months after stroke but are sometimes identified after they have been discharged from therapy. These findings and the fact that participants improved due to practice and were benefitting from compensatory technologies offer further support for the development of therapies and technologies that could support self‐management for people with aphasia (Nichol et al., 2019).

Participants emphasized the difficulties that they had with writing, in terms of how exhausting and difficult it felt and their negative perceptions of their written output. Whereas the writing rehabilitation literature and writing assessments have traditionally focused on accuracy, some of the participants’ concerns were related to other aspects of writing, including efficiency, which has been shown to be a challenge for people with aphasia (Johansson‐Malmeling et al., 2021). This suggests that writing therapies and assessments should go beyond a focus on spelling accuracy.

Many barriers to writing were identified. It is perhaps unsurprising that other stroke‐related symptoms, such as fatigue, cognitive, visual and motor difficulties, and depression impacted on participants’ writing. Depression, which occurs in around 62–70% of people with aphasia (Lincoln et al., 2011; Worrall et al., 2016), has been associated with poorer post‐stroke recovery (Donnellan et al., 2010), and cognitive impairments have been associated with poorer outcomes of word‐retrieval therapies (Lambon Ralph et al., 2010). The participants’ understanding of the barriers impacting on their writing demonstrates the importance of interviewing and person‐centred goal setting to guide tailored approaches to intervention (Elston et al., 2021; Parr, 1995; Thomson et al., 2018).

### Importance of writing for fulfilling adult social roles

Writing skills were considered to be important to participants, particularly for communication with friends and family. They described the importance of writing in relation to social roles and responsibilities. This reflected findings by Barton and Padmore (1991) who found that when people (without aphasia) discussed their everyday writing practices, they tended to refer to their social roles, for example, referring to themselves as parents or friends, and the differing demands that each role put on them. For some of the participants in this study, writing had become even more necessary due to reduced spoken language ability, as they relied on writing skills to support verbal conversations and found it easier to text message than to speak on the phone. For example, in writing messages there is more time to consider, plan and edit than there is in verbal conversations where there can be additional pressure (Mortenson, 2004). This mirrors Kjellen et al.’s (2017) findings that participants were motivated to write so that they could communicate with people, as well as Parr's (1995) findings that writing practices reflect participants’ social roles which may change due the individual's language impairment and changing roles as a consequence of aphasia.

Writing impairments restricted the participants from carrying out activities, social roles and participating in society, as they had done previously. This reduced participation could result in reduced power to maintain relationships and social networks and to interact more formally with organizations, and therefore an increased risk of being socially excluded (Kjellen et al., 2017; Mortenson, 2004; Parr, 2007). Participants’ writing difficulties sometimes caused embarrassment and contributed to a negative self‐concept of being an adult who cannot spell, which may be exacerbated by the lack of public awareness of aphasia (Code et al., 2016) and echoes the results of previous studies (Behrns et al., 2009; Knollman‐Porter et al., 2015; Kjellen et al., 2017). Kjellen et al.’s (2017) participants felt loss, frustration, annoyance and disappointment due to their reading and writing difficulties, and their self‐image changed dramatically. Aphasia has been associated with ‘identity theft’ (Shadden, 2005), meaning that it can lead to significant changes to personal and social identity (Shadden, 2005) as identity is shaped by relationships which are formed through communication (Taubner et al., 2020). This study has shown that a disruption to one's identity may result from not just spoken, but also written language difficulties and restrictions in written communication, due to the societal expectation that adults can write. The association of poor writing skills and perceptions of a child‐like state, or of lacking adult competence, was also coupled with some hesitancy about use of compensatory technology with descriptions of ‘cheating’, rather than active and pragmatic use of readily supportive technology.

Participants felt that there was less opportunity for writing since their stroke, due to a change in roles and responsibilities, for example, no longer working. It might be expected that these changes to participants’ circumstances would all be negative, as change is often associated with loss (Parr, 1995), but there were a range of feelings about this with one participant feeling relieved that she no longer needs to write constantly, as she associated writing with the stress and exhaustion that she had experienced in her job. A further reason for reduced opportunity for writing was that the participants had fewer people to write to than before. Cruice et al. (2006) found that participants without aphasia had, on average, nine more social contacts than participants with aphasia, and that participants with aphasia were less satisfied with their social activities than non‐aphasic peers. For some participants, family members took on the role of domestic tasks, such as paying the bills or filling out forms, which reduced their independence. People with aphasia may, therefore, be forced to adopt a passive role with domestic tasks due to their writing difficulties, in a similar way that they may take on a passive role within conversations with their partners due to spoken language difficulties (Lock et al., 2001). Independence has been identified as an important factor in living successfully with aphasia. Brown et al. (2010), for example, found that people with aphasia become independent by participating in meaningful activities. Furthermore, there is a likelihood that lack of engagement in writing reinforces a lack of practice in the component skills in writing, potentially leading to learned non‐use of writing as a dynamic cognitive–linguistic–motor process (Wolf et al., 1989). This dependence may also affect identity as, according to Shadden (2005), identity renegotiation is dependent on social contexts and social others.

The reasons for the reduced opportunities for writing reflect Parr's (1995: 234) findings that literacy practices are influenced by a variety of factors, which among others include ‘the reorganization of domestic, work, leisure and social roles and purposes’ and ‘patterns of dependence and interdependence and associated levels of autonomy’. These findings also emphasize that writing is a sociocultural practice, which reflects an individual's social roles and domains and constantly changes through life as their social roles and networks change (Barton & Padmore, 1991; Kjellen et al., 2017; Mortenson, 2004). As Mortenson (2004) explains, writing is a social behaviour, which should be considered in relation to an individual's personal identity.

Although, for most participants it had been many years since their stroke, they described an array of individual goals related to writing. These findings on writing goals relate to the work by Knollman‐Porter et al. (2015) on reading in aphasia. They discovered that participants continued to be motivated to read even though they found this difficult. This optimism and motivation to keep practising and participating in meaningful activities has positive implications for rehabilitation and engagement in social activities (Dalemans et al., 2010; Grohn et al., 2014).

### Strengths and limitations

This study included a diverse range of participants in terms of age, education, and aphasia and dysgraphia symptoms and severities, and the interview schedule was piloted to ensure that it was accessible to the participants with aphasia. Transcription and analysis included multi‐modality communication, including writing, gestures, pointing and facial expressions. This type of approach to the interviews and analytical process is important for including people with aphasia (Luck & Rose, 2007). The researcher took a reflexive approach throughout the study in accounting for her own role. In‐depth analysis of interviews was conducted using inductive reflexive thematic analysis guided by Braun and Clarke (2006) and an audit trail was kept. This study provided a clear account of procedures and an in‐depth analytical narrative of the findings with inclusion of multiple quotations to demonstrate each theme. Negative cases were sought in the process of reviewing themes.

The rigour of this study could have been improved through the use of field notes, a reflective journal and participant validation (O'Brien et al., 2014; Tong et al., 2007). It is important to acknowledge that the findings are limited to the context of this study. The participants were a self‐selected sample of participants who demonstrated through participating in the previous study that they were all motivated and interested in improving their writing. Their participation in the previous intervention study will likely have influenced their answers in the interviews. They were between two and 20 years post‐stroke and in most cases had identified their writing needs and goals after speech and language therapy had finished. These factors reduce the transferability of findings to people with aphasia receiving speech and language therapy as not all will have writing goals within early post‐stroke rehabilitation. The researcher is a speech and language therapist who had experience of working with the participants on their writing, as the researcher carrying out the intervention in the previous study, which will have influenced her interpretation of the findings. Finally, it is important to acknowledge that there have been advances in technology and therefore differences in writing experiences since these interviews were conducted in 2014.

### Clinical implications

The participants emphasized the importance of writing for fulfilling adult social roles and were highly motivated to keep working towards specific writing goals, which suggests that for certain clients, writing goals should be prioritized in speech and language therapy, particularly as improved writing could improve social interaction, participation in society and self‐esteem and confidence. The findings demonstrate the importance of interviewing to support person‐centred goal setting in aphasia rehabilitation (Elston et al., 2021; Thomson et al., 2018) as the participants were able to provide detailed descriptions of their activities, goals, difficulties, facilitators, and barriers in writing. Informal assessment of a range of functional writing tasks is necessary to gather information on the individual's strengths and difficulties and to set appropriate person‐centred goals.

Management should capitalize on existing strategies and support identified by the individual. In addition to impairment‐based spelling therapy, self‐management approaches should be explored, such as computerized therapy to support spelling practice, and assistive technologies to compensate for writing difficulties and provide opportunities to participate in everyday activities. As the participants emphasized the importance of support from family members and friends, it would be helpful for them to be involved in intervention, which could include identifying opportunities for completing everyday writing tasks. Furthermore, there seems to be an important role for group therapy which can give people with aphasia the opportunity to share strategies. Writing groups could serve as a useful therapeutic vehicle to provide a bridge from non‐writing after a stroke to independent social use of writing. The range of perceived difficulties and goals provided evidence for assessment and therapy that is tailored to the individual's needs and activities. Therapy should include meaningful activities that relate to functional everyday writing.

## CONCLUSIONS

This study has contributed to the aphasia literature by building upon the findings of previous studies and providing further understanding of the experiences of people with mild to severe aphasia and writing difficulties. This has provided novel insights into the participants’ perceptions of improvements over time and facilitators and barriers to improvements. The findings have also shown the value that participants place on their writing for communication and fulfilling adult social roles and the impact of their dysgraphia on participation in society, self‐esteem and confidence. Participants had different, though in some cases more personally important, purposes for writing, compared with before their stroke, and were still motivated to work towards writing goals at the time of the study. This study has reinforced that people with a range of types and severities of aphasia can participate in interviews if appropriate support is provided, and that they can contribute important insights.

## CONFLICTS OF INTEREST

The authors report no conflicts of interest. The authors alone are responsible for the content and writing of the paper.

## FUNDING

The research was conducted as part of the first author's PhD, which was funded by the Economic and Social Research Council (ESRC) (award number ES/I020233/1).

## Supporting information

Table A1

## Data Availability

The data that support the findings of this study are available from the corresponding author upon reasonable request.
