# Supplementary material for: ‘I think writing is everything’: An exploration of the writing experiences of people with aphasia
Source: Int J Lang Commun Disord. 2022 Aug 5;57(6):1381–98. doi: 10.1111/1460-6984.12762 (PMC9805004; doi:10.1111/1460-6984.12762)
Supplement: Supplementary file 1 — Table A1 [file JLCD-57-1381-s001.docx]

**Completed COREQ (Consolidated criteria for reporting qualitative studies) Checklist (Tong et al., 2007)**

| **Domain 1: Research team and reflexivity**  Personal characteristics | | |
| --- | --- | --- |
| 1. Interviewer/facilitator | Which author/s conducted the interview or focus group? | LT conducted the interviews |
| 2. Credentials | What were the researcher’s credentials? E.g. PhD, MD | BSc (Hons) Speech and Language Therapy  MRes Psychology  PhD Psychology |
| 3. Occupation | What was their occupation at the time of the study? | Speech and Language Therapist  PhD Student |
| 4. Gender | Was the researcher male or female? | Female |
| 5. Experience and training | What experience or training did the researcher have? | LT was new to qualitative research but had guidance from her supervisor (PC) and support from an academic (MC) external to the project |
| Relationship with participants | | |
| 6. Relationship established | Was a relationship established prior to study commencement? | All of the participants had taken part in the researcher’s previous study, evaluating assistive writing technologies. 6 of the participants had taken part in another previous spelling therapy study with the researcher. |
| 7. Participant knowledge of the interviewer | What did the participants know about the researcher? e.g. personal goals, reasons for doing the research | Participants knew that LT was a speech and language therapist and that her aims were to evaluate the effects writing interventions and to explore participants’ experiences of literacy and interventions |
| 8. Interviewer characteristics | What characteristics were reported about the interviewer/facilitator? e.g. Bias, assumptions, reasons and interests in the research topic | LT’s background as an SLT and knowledge of the participants have been reported in the paper. It has been stated that the data were viewed through the lens of a speech and language therapist who had experienced the participants’ progress and challenges and were invested in their writing improvements. |
| **Domain 2: study design**  Theoretical framework | | |
| 9. Methodological orientation and Theory | What methodological orientation was stated to underpin the study? e.g. grounded theory, discourse analysis, ethnography, phenomenology, content analysis | A constructivist paradigm was adapted.  Data were analysed using inductive reflexive thematic analysis |
| Participant selection | | |
| 10. Sampling | How were participants selected? e.g. purposive, convenience, consecutive, snowball | Purposive sampling. Participants had taken part in the previous writing therapy studies. They were recruited for the previous studies from stroke support groups or through other previous studies, where they had expressed an interest in volunteering for further studies. |
| 11. Method of approach | How were participants approached? e.g. face-to-face, telephone, mail, email | Participants were approached face to face in final sessions of the previous study. They were given a participant information sheet and were told about the study verbally and had an opportunity to ask questions and to consider whether they would like to participate, before being asked to sign a consent form. |
| 12. Sample size | How many participants were in the study? | Eight participants |
| 13. Non-participation | How many people refused to participate or dropped out? Reasons? Setting | None of the participants who were asked to take part refused |
| 14. Setting of data collection | Where was the data collected? e.g. home, clinic, workplace | The interviews took place in participants’ homes |
| 15. Presence of non-participants | Was anyone else present besides the participants and researchers? | Nobody else was present. Participants’ spouses were at home but did not participate in the interviews and were not in the same room |
| 16. Description of sample | What are the important characteristics of the sample? e.g. demographic data, date | Years since stroke: All participants had had a stroke between 1 and 20 years previously  Age: 47-80 years  Education: 9-16 years  Gender: 5 female; 3 male  All participants had aphasia and acquired writing difficulties. |
| Data collection | | |
| 17. Interview guide | Were questions, prompts, guides provided by the authors? Was it pilot tested? | A topic guide with questions and prompts was developed and used for interviews. This was pilot tested on one participant but no changes were made. The topic guide has been provided as Appendix 1. |
| 18. Repeat interviews | Were repeat interviews carried out? If yes, how many? | No repeat interviews were carried out |
| 19. Audio/visual recording | Did the research use audio or visual recording to collect the data? | The interview sessions were video recorded and gestures and writing were included in the interview transcripts |
| 20. Field notes | Were field notes made during and/or after the interview or focus group? | No |
| 21. Duration | What was the duration of the interviews or focus group? | Interviews were 60-90 minutes long |
| 22. Data saturation | Was data saturation discussed? | Data saturation was not discussed |
| 23. Transcripts returned | Were transcripts returned to participants for comment and/or correction? | Transcripts were not returned to participants for comment or correction. |
| Domain 3: analysis and findings  Data analysis | | |
| 24. Number of data coders | How many data coders coded the data? | Only LT coded the data |
| 25. Description of the coding tree | Did authors provide a description of the coding tree? | No coding tree was used. Examples have been provided in Table 3 of interview excepts and corresponding codes, subthemes and themes. |
| 26. Derivation of themes | Were themes identified in advance or derived from the data? | Themes were derived from the data |
| 27. Software | What software, if applicable, was used to manage the data? | No software was used |
| 28. Participant checking | Did participants provide feedback on the findings? | Participants were not asked to provide feedback on the findings |
| Reporting | | |
| 29. Quotations presented | Were participant quotations presented to illustrate the themes / findings? Was each quotation identified? e.g. participant number | Yes, participant quotations have been presented and each participant has been labelled with a participant number |
| 30. Data and findings consistent | Was there consistency between the data presented and the findings? | An attempt has been made to report the data and findings clearly and consistently |
| 31. Clarity of major themes | Were major themes clearly presented in the findings? | Major themes have been presented clearly |
| 32. Clarity of minor themes | Is there a description of diverse cases or discussion of minor themes? | Subthemes have been presented clearly and negative examples have been included |

Tong, A., Sainsbury, P. & Craig, J. (2007). Consolidated criteria for reporting qualitative research (COREQ): a 32-item checklist for interviews and focus groups. *International Journal for Quality in Health Care, 19*(6), 349 –357.
